# The Basilar Artery International Cooperation Study (BASICS): study protocol for a randomised controlled trial

**DOI:** 10.1186/1745-6215-14-200

**Published:** 2013-07-08

**Authors:** Erik JRJ van der Hoeven, Wouter J Schonewille, Jan Albert Vos, Ale Algra, Heinrich J Audebert, Eivind Berge, Alfonso Ciccone, Mikael Mazighi, Patrik Michel, Keith W Muir, Víctor Obach, Volker Puetz, Cristanne AC Wijman, Andrea Zini, Jaap L Kappelle

**Affiliations:** 1 Department of Radiology, St. Antonius Hospital, PO Box 2500, Nieuwegein, EM, 3430, The Netherlands; 2 Department of Neurology, St. Antonius Hospital, PO Box 2500, Nieuwegein, EM, 3430, The Netherlands; 3 Department of Neurology and Neurosurgery, Rudolf Magnus Institute for Neuroscience, University Medical Center Utrecht, HP G 03.228, PO Box 85500, Utrecht, GA, 3508, The Netherlands; 4 Julius Center for health Sciences and Patient Care, University Medical Center Utrecht, HP STR 6.131, PO Box 85500, Utrecht, GA, 3508, The Netherlands; 5 Center for Stroke Research Berlin and Department of Neurology, Charité Universitätsmedizin Berlin, Campus Benjamin Franklin, Hindenburgdamm 30, Berlin, 12200, Germany; 6 Department of Internal Medicine, Oslo University Hospital Ullevål, Oslo, NO-0407, Norway; 7 Department of Neurology and Stroke Unit, Carlo Poma Hospital, Strada Lago Paiolo 10, Mantua, 46100, Italy; 8 Department of Neurology and Stroke Centre, Bichat University Hospital, 46 rue henri Huchard, Paris, 75018, France; 9 Department of Clinical Neurosciences CHUV, BH/13/204 Rue du Bugnon 46, Lausanne, CH-1011, Switzerland; 10 Institute of Neurosciences & Psychology, Southern General Hospital, University of Glasgow, Glasgow, G51 4TF, UK; 11 Comprehensive Stroke Center, Hospital Clinic de Barcelona, Villarroel 170, Barcelona, 08036, Spain; 12 Department of Neurology, Dresden University Stroke Centre, Technical University Dresden, Fetscherstraße 74, Dresden, 01307, Germany; 13 Stanford University Medical Center, Stanford Stroke Center, 780 Welch Road, Suite 205, Palo Alto, CA, 94304, USA; 14 Stroke Unit, Department of Neuroscience, University of Modena and Reggio Emilia, St. Agostino-Estense Hospital, Modena, AUSL, Italy

**Keywords:** Basilar artery occlusion, Basilar artery thrombosis, Intra-arterial treatment, Intravenous thrombolysis, Stroke

## Abstract

**Background:**

Despite recent advances in acute stroke treatment, basilar artery occlusion (BAO) is associated with a death or disability rate of close to 70%. Randomised trials have shown the safety and efficacy of intravenous thrombolysis (IVT) given within 4.5 h and have shown promising results of intra-arterial thrombolysis given within 6 h of symptom onset of acute ischaemic stroke, but these results do not directly apply to patients with an acute BAO because only few, if any, of these patients were included in randomised acute stroke trials.

Recently the results of the Basilar Artery International Cooperation Study (BASICS), a prospective registry of patients with acute symptomatic BAO challenged the often-held assumption that intra-arterial treatment (IAT) is superior to IVT. Our observations in the BASICS registry underscore that we continue to lack a proven treatment modality for patients with an acute BAO and that current clinical practice varies widely.

**Design:**

BASICS is a randomised controlled, multicentre, open label, phase III intervention trial with blinded outcome assessment, investigating the efficacy and safety of additional IAT after IVT in patients with BAO. The trial targets to include 750 patients, aged 18 to 85 years, with CT angiography or MR angiography confirmed BAO treated with IVT. Patients will be randomised between additional IAT followed by optimal medical care versus optimal medical care alone. IVT has to be initiated within 4.5 h from estimated time of BAO and IAT within 6 h. The primary outcome parameter will be favourable outcome at day 90 defined as a modified Rankin Scale score of 0–3.

**Discussion:**

The BASICS registry was observational and has all the limitations of a non-randomised study. As the IAT approach becomes increasingly available and frequently utilised an adequately powered randomised controlled phase III trial investigating the added value of this therapy in patients with an acute symptomatic BAO is needed (clinicaltrials.gov: NCT01717755).

## Background

Stroke is the leading cause of disability in developed countries [1]. Posterior circulation stroke accounts for about 20% of all ischaemic strokes. The basilar artery is the main vessel of the posterior circulation that supplies most of the brainstem and occipital lobes, and part of the cerebellum and thalami. Basilar artery occlusion (BAO) can cause many symptoms such as isolated cranial nerve palsies or hemiplegia, but also a locked-in state or coma. Despite recent advances in acute stroke treatment BAO is associated with death or disability rate of close to 70% [2].

Randomised trials have shown the safety and efficacy of intravenous thrombolysis given within 4.5 h and promising results of intra-arterial thrombolysis given within 6 h of symptom onset of acute ischaemic stroke [3-8]. Unfortunately these results do not directly apply to patients with an acute BAO because only few, if any, of these patients were included in randomised acute stroke trials. As yet, BAO has not been studied in isolation in randomised clinical trials. Patients with BAO only represent about 5% of all thrombolysed stroke patients [9,10]. We are aware of only one attempt to perform a randomised treatment trial in patients with an acute BAO, which was terminated prematurely because of poor recruitment [11]. Case series of patients with BAO found similar outcomes in patients treated with antithrombotic therapy, intravenous thrombolysis (IVT) or intra-arterial treatment (IAT) [11,12].

Recently our study group reported the results of the BASICS registry, a prospective registry of patients with an acute BAO [2]. We were not able to identify a statistically significant superior treatment strategy. However the inclusion of >600 patients in the registry over a 5-year period suggests that the performance of a randomised trial in patients with BAO is feasible.

Our observations in the BASICS registry underscore that we continue to lack a proven treatment modality for patients with an acute BAO and that current clinical practice varies widely. Furthermore, the often-held assumption that IAT is superior to IVT in patients with an acute symptomatic BAO is challenged by our data. Although recanalisation rates are consistently higher after IAT as compared to IVT in observational studies, this was not consistently accompanied by improved outcome [13,14].

The BASICS registry was observational and has all the limitations of a non-randomised study. Reasons for clinicians to select a specific treatment option are more complex than can be captured in the scope of a prospective registry. Multivariable analyses can never adjust completely for systematic differences between treatment groups. A bias towards a more aggressive treatment approach in patients who were thought to have a worse prognosis may have influenced the outcome in the IAT group and relinquishing both IVT and IAT in patients with a severe deficit may have been an expression of a more palliative approach. Crossover to another treatment group because of clinical worsening or lack of treatment response was not taken into account. There may have been unmeasured variables relevant to outcome that were imbalanced between groups.

As the IAT approach becomes increasingly available and frequently utilized an adequately powered large randomised controlled phase III trial investigating the added value of this therapy in patients with an acute symptomatic BAO is needed.

## Methods

BASICS is a multicentre, open label, randomised, controlled, phase III trial comparing optimal medical care with best intra-arterial therapy in patients with BAO who were treated with intravenous thrombolysis. A total of 750 patients will be included. Follow-up will continue until 1 year after inclusion of the last patient.

### Enrollment criteria

Patients can be enrolled in the study if the following criteria have been met:

1. symptoms and signs compatible with ischaemia in the basilar artery territory and an National Institutes of Health Stroke Scale (NIHSS) ≥10 at time of randomisation;

2. BAO confirmed by CTA or MRA;

3. aged 18 to 85 years;

4. initiation of IV rt-PA within 4.5 h of estimated time of BAO. Estimated time of BAO is defined as time of onset of acute symptoms consistent with the clinical diagnosis of basilar artery occlusion or if not known last time patient was seen normal prior to onset of these symptoms, hence time from symptom onset can be considerably longer than 4.5 h;

5. initiation of IA therapy should be feasible within 6 h of estimated time of BAO;

6. informed consent.

Patients will be excluded from the study in case of:

1. pre-existing dependency with a modified Rankin scale (mRS) ≥3;

2. female of childbearing potential who is known to be pregnant or lactating or who has a positive pregnancy test on admission;

3. need for haemodialysis or peritoneal dialysis;

4. other serious, advanced or terminal illness;

5. any other condition that the investigator feels would pose a significant hazard to the patient if IA therapy is initiated;

6. current participation in another research drug treatment protocol (patient cannot start another experimental agent until after 90 days);

7. lesion consistent with haemorrhage of any degree on neuroimaging;

8. significant cerebellar mass effect or acute hydrocephalus on neuroimaging;

9. bilateral extended brainstem ischaemia on neuroimaging.

### Study procedures

Based on the experience in the BASICS registry an estimated 40% to 50% of patients will present in community hospitals with subsequent referral to an intervention centre. Community hospitals should be encouraged to initiate IVT prior to transfer according to the ‘drip and ship’ principle. Intubation prior to transfer should be strongly encouraged in any subject deemed unstable or at high risk of aspiration. If sedation is needed, short acting drugs, like propofol (di-isopropylfenol) should be given to avoid interference with the neurological examination upon arrival at the intervention centre. A diagnostic neuroimaging screening with CT/CTA or MRI/MRA confirming the presence of BAO and the absence of imaging exclusion criteria, and a NIHSS of 10 or more will be used to identify patients eligible for the trial.

In case of an increase in NIHSS by ≥5 points during transfer and in any comatose subject a repeat CT scan of the brain should be performed prior to randomisation to exclude intracranial hemorrhage. In those patients in whom BAO is assessed prior to transfer a repeat CTA should be performed prior to randomisation in the intervention centre to reassess basilar artery patency in case of an improvement in NIHSS by ≥5 points during transfer or a time delay beyond 2 h after initial confirmation of BAO and in any comatose subject.

### Randomisation

After obtaining informed consent patients will be randomised into one of the two treatment arms. Patients are randomised by a secure link to a central randomisation database. Randomisation will be stratified for stroke severity (NIHSS score <20 versus ≥20), for centre and for time of symptom onset (within 4.5 h of symptom onset versus beyond 4.5 h of symptom onset, but within 4.5 h of estimated time of BAO).

### Registry of patients with BAO who are not randomised

To evaluate a possible selection bias of patients included in the trial, participating centres are obliged to enter all patients with acute symptomatic BAO presenting at their centre who are treated with IVT or IAT but who are not randomised, in a registry. Data are collected on patient characteristics, time to treatment, eligibility, reason for non-inclusion and type of treatment.

### Treatment

One of the guiding principles of the BASICS trial is rapid initiation of thrombolytic therapy to an eligible subject to provide maximal benefit. To minimize any delay in the administration of a proven effective therapy (that is, IV rt-PA), the standard dose of open-label IV rt-PA (0.9 mg/kg; 90 mg maximum) is initiated prior to enrolment and randomisation in the trial if standard eligibility criteria are met.

Patients treated with IVT within 4.5 h of first symptom onset, and those who are treated beyond 4.5 h of first symptom onset, but within 4.5 h of estimated time of BAO, will be regarded as two pre-specified subgroups for secondary analysis. In patients treated beyond 4.5 h of symptom onset, informed consent needs to be obtained prior to initiation of IVT.

IA therapy has to be initiated within 6 h of estimated time of BAO. Endovascular treatment will be performed by an experienced interventional radiologist with a track record of at least 10 intra-arterial interventions both in the middle cerebral and basilar artery in the last 2 years. If an appropriate thrombus or residual stenosis is identified, the choice of IA strategy will be made by the treating neurointerventionalist.

### Objectives

The primary objective of the trial is to evaluate the efficacy of additional IAT in patients with BAO treated with IVT, in terms of favourable outcome at 90 days, defined as a modified Rankin score of 0–3.

Secondary analysis will compare outcome in the following pre-defined subgroups: patients with a baseline NIHSS of 10–19, and those with a baseline NIHSS of ≥20.

Patients treated with IVT within 4.5 h of first symptom onset, and those treated beyond 4.5 h of first symptom onset within 4.5 h of estimated time of BAO.

Secondary objectives are safety evaluation of a combined IV/IA approach compared with IV rt-PA alone, evaluation of the safety and efficacy of mechanical devices as part of a combined IV/IA approach and evaluation of efficacy of a combined IV/IA approach compared with IV rt-PA alone in terms of a favourable outcome on other clinical and radiological measures. Other clinical and radiological measures for evaluation of efficacy will be: (1) Excellent outcome defined as a mRS of 0–2 at day 90 and 1 year; (2) mRS - not dichotomized at day 90 and 1 year; (3) EQ-5D at day 90 and 1 year; (4) an improved early response to treatment as determined by a reduction in NIHSS by 5 points or more at 24 h; (5) a CT or MR angiography assessment of basilar artery patency at 24 h, and (6) the extent of cerebral infarction as measured by the pc-ASPECTS score on NCCT (non-contrast CT) or MRI at 24 h. The primary measures for evaluation of safety will be symptomatic intracranial haemorrhage or intracranial haemorrhage contributing to patients’ death as determined by the study safety committee confirmed on neuroimaging within 3 days of treatment initiation (CT or MRI), or overall mortality at 90 days.

### Follow-up

Length of follow-up will be 1 year with a blinded exam at day 90 (mRS, EQ-5D) and telephone surveys at 30 days (mRS) and 1 year (mRS, EQ-5D).

### Statistical considerations

#### Power calculation

Assuming an absolute increase of 10% of favourable outcome at 90 days by additional IA therapy compared to optimal medical care alone, we calculated that 712 patients would be needed. This calculation was based on a type 1 error of 5%, a type 2 error of 20%, and a presumed incidence of the primary outcome event of 30% in the group treated with optimal medical care. This latter incidence was based on data of the BASICS registry study [2]. Based on these assumptions the trial would yield a risk ratio of 1.33 with a 95% confidence interval of 1.09 to 1.63, that is, a relative increase of 33% more patients with a favourable outcome with additional IA therapy. The sample size formula used originated from the standard work on clinical trials by SJ Pocock [15]. To account for potential dropout a target of 750 patients was set.

#### Data analysis

Continuous data will be summarised with means and standard deviations. For count data percentages will be given.

The primary aim of the univariable analysis is to compare the proportion of patients with a favourable outcome at 90 days between the two treatment groups. For this purpose a risk ratio with corresponding 95% CI will be calculated. The analyses will be based on the intention-to-treat principle.

Multivariable analyses will only be carried out if important incomparability is detected between the two treatment groups. In that case risk ratios will be calculated that are adjusted for the variables that show baseline imbalance. To this end Poisson regression will be used, similar to that used in the BASICS registry study [2].

### Safety reporting

An adverse event (AE) is any unfavourable and unintended sign, symptom, or disease occurring during the follow-up period of the study. Adverse events occurring after randomisation will be recorded on the adverse event page of the CRF. A serious adverse event (SAE) is defined as any adverse event that results in: (1) death; (2) a life-threatening condition; (3) inpatient hospitalisation or prolongation of existing hospitalisation; or (4) persistent or significant disability/incapacity.

Adverse reactions are all untoward and unintended responses to the investigational treatment related to any dose or device used.

Serious unexpected adverse reactions (SUSARs) are adverse reactions, of which the nature, or severity, is not consistent with the applicable product information.

All SAEs and SUSARS will be reported to and collected by the data coordination unit. An Internal Safety Committee will review safety data on an ongoing basis, including monitoring the trend in serious adverse outcome events and submitting reports to regulatory agencies and the data safety monitoring board (DSMB).

### Monitoring

An independent DSMB will monitor the trial. For efficacy a symmetrical two-sided stopping rule will be used. The size of the trial is based on the assumption of a 10% absolute increase of the proportion of patients with a favourable outcome treated with additional IA therapy as compared with optimal medical care alone. If the observed benefit is ‘clearly’ larger or if optimal medical care appears to be better than additional IA therapy early termination of the trial may be recommended. A restricted procedure will be used with alpha equal to 0.05 and a power of 0.80 [16].

Safety will be monitored as follows. The BASICS registry observed that the risk of symptomatic intracranial haemorrhage (sICH) in patients treated with IA therapy was 14% (95% CI 10-18%) and 7% (95%CI 3-11%) in those treated with IVT only [2]. A more than two-fold excess of symptomatic intracranial haemorrhage in the group treated with additional IA therapy as compared with maximum supportive treatment may therefore be considered as problematic. However, symptomatic intracranial haemorrhage is a contributing component of the primary outcome and hence is weighed during monitoring of this outcome. A higher than expected sICH rate in the absence of a significant difference in functional outcome may therefore lead to a decision to put the trial on hold to analyse the reason for this higher sICH rate and the need to change treatment recommendations such a adjustments in the dose of thrombolytics used or the use of specific devises.

The BASICS Trial Office will put an active follow-up system into effect, such that for each patient 90-day follow-up data and those on the occurrence of symptomatic intracranial haemorrhage are obtained without delay. The first interim analysis will be performed at the moment the 3-month follow-up data are available on the first 10 patients randomised. Every 4 months thereafter the DSMB will be given the latest follow-up data and will advise the steering committee about the continuation of the trial based on these data. Thus a sequential monitoring process is installed based on the procedures described by Whitehead in 1997 [16]. For this purpose the program PEST 4 will be used. The recommendation on the continuation will be based on: (1) stopping rules as described above; and (2) the most recent information from medical literature or congresses in the field of cerebrovascular disease.

### Ethical considerations

The study will be conducted according to the principles of the Declaration of Helsinki (http://www.wma.net21-10-2008) and in accordance with the Dutch Medical Research Involving Human Subjects Act (WMO). The BASICS trial was approved by the ethics committee of the St. Antonius Hospital on 20 January 2011 and is registered under number R-10.39A. National ethical approval was given by the Central Commission of Human Research (CCMO) on 21 December 2010 and is registered under number NL33550.100.10. Approval by the local medical ethical review board is required for each participating centre before start of patient inclusion.

Patients or their legal representatives will be informed about the trial by their treating neurologist or neurology resident who will also obtain informed consent. In acute situations or if the patient is incapable to give written informed consent oral informed consent may be obtained. In case of oral informed consent a witness (for example, family or nurse) should be present when the information is presented to the patient or patient representative. A written summary that describes the essential information will be presented to the patient or patient representative. The witness, and responsible neurologist or neurology resident will sign this document.

In case of a subject with severe decrease of consciousness (along with national legislations), informed consent can be obtained from the patient’s proxy in person or by telephone as long as the identity of the proxy can be confirmed. If approved by the local ethical review board an independent physician can sign informed consent. Community hospitals are encouraged to obtain informed preliminary consent for trial participation of the subject or his proxy prior to transfer.

In patients who are not eligible for standard IVT because IVT cannot be initiated within 4.5 h of symptom onset but who are treated within 4.5 h of estimated time of BAO informed consent has to be obtained prior to IVT. In those centres where CTA or MRA is not part of the standard acute stroke work-up informed consent has to be obtained prior to CTA or MRA.

### Publication of the trial results

The trial results will be published by the members of the Executive and Steering Committee, on behalf of the investigators.

## Discussion

There are several factors that distinguish patients with BAO from those with middle cerebral artery (MCA) occlusion that may warrant a different treatment approach:

*Severity of deficit:* previous studies have suggested a greater benefit of IA therapy in patients with a severe deficit.

*High poor outcome rate:* because of a higher poor outcome rate, patients with BAO have more to gain.

*Collateral flow:* the basilar artery not only receives collateral flow through cortical cerebellar branches, comparable to the cortical hemispheric branches of the MCA, but also by retrograde filling by the anterior circulation through the posterior communicating arteries as part of the circle of Willis. IVT may be more effective in the presence of good collateral flow, by attacking the thrombus from both sides.

*Time window for treatment:* the BASICS registry data suggest the presence of a longer time window from symptom onset to time of treatment. The PROACT studies used a limit of time from symptom onset to time of treatment of 6 h [4,17]. The IMS III study used a 5-h time window from symptom onset to initiation of IAT [18]. The BASICS trial uses a 6-h time window from estimated time of BAO to initiation of IA treatment. The BASICS registry used the estimated time of symptom onset consistent with the clinical diagnosis of BAO to treatment rather than the more commonly used time of onset of any symptoms to treatment. Previous studies have shown that BAO is preceded by prodromal symptoms in >60% of patients [19,20]. Most of these patients would be excluded from a potential trial using the time of onset of first symptom <4.5 h to treatment as an inclusion criterion. We believe that the results from the BASICS registry support the use of the estimated time of BAO rather than using the time of onset of any symptom to treatment as an inclusion criterion for the BASICS trial.

*IVT* versus *IVT/IAT comparison:* IV thrombolysis is the current standard of care in patients presenting with acute ischaemic stroke with a proven safety and efficacy and therefore should be regarded as the current ‘Gold Standard’ with which potential new treatment strategies should be compared.

The use of an IVT only arm in a trial of patients with acute symptomatic BAO is supported by the results of the BASICS registry in which no significant difference was found between IVT or IAT treated patients with a severe deficit [2].

The performance of a trial comparing IVT alone vs. IAT alone in patients with BAO does not seem feasible nor ethical. Referral of a patient to an intervention centre for randomisation between IVT vs. IAT alone would mean delaying the initiation of a treatment which is of proven benefit - whereas there is convincing evidence for the principle of ‘time is brain’, also in patients with BAO [21,22]. The number of patients with BAO presenting directly to an intervention centre will be too limited. Patients with BAO only represent an approximate 5% of all IVT eligible patients, and only 40% of patients in the BASICS registry where admitted directly (without referral) to an intervention centre.

In order to include a sufficient number of patients the BASICS trial will therefore mainly depend on the inclusion of patients referred from non-intervention community hospitals. The non-intervention hospitals are encouraged to start IVT without delay before sending the patient with the clinical diagnosis of BAO to the intervention centre.

A combined IV and IA approach to acute ischaemic stroke therapy was designed to offer rapid initiation of IV rt-PA, followed by additional titrated local IA therapy, to patients with moderate-to-severe strokes (NIHSS ≥10). The goal was to achieve higher rates of early, successful reperfusion in a widely accessible manner. This approach has been tested in clinical trials of >200 patients, starting with the Emergency Management of Stroke (EMS) pilot trial from 1995 to 1996, followed by the Interventional Management of Stroke (IMS) I trial in 2001, the IMS II trial from 2003 to 2006, and several additional cohorts [23-25]. The data from EMS and IMS show that the combined approach to recanalisation may be more effective than standard IV rt-PA alone for moderate-to-severe (NIHSS ≥10) strokes, while maintaining a similar safety profile. The recently published IMS III trial data did not show a significant difference in outcome comparing IV rt-PA with IV rt-PA followed by IAT in patients with a NIHSS of 8 or greater treated within 3 h [26]. Few patients with BAO were included, about 2% in both treatment arms. Furthermore, few patients had radiologic confirmation of occlusion and most patients in the IA arm were treated with IA thrombolysis or first generation thrombectomy devices, much less effective than the currently used stentretrievers [27,28].

### IVT arm

The 4.5-h time window is based on the results of the ECASS III study [7]. A time window of 0–4.5 h from symptom onset to treatment in patients with acute ischaemic stroke is widely accepted. The BASICS registry results show the safety of using a 0 to 4.5-h time window from estimated time of occlusion to treatment in patients with acute BAO [2].

### IVT + IAT arm

Based on the results of the PROACT studies the 6-h time window for IA thrombolysis in patients with MCA occlusion is widely accepted [4,17].

A case series of 69 patients treated with IA thrombolysis (urokinase, reteplase or alteplase) following full dose IVT showed the safety of full dose IVT followed by IA thrombolysis. Symptomatic haemorrhage occurred in four out of 69 (5.8%) patients [29].

The MERCI studies suggested safety of mechanical thrombectomy up to 8 h from symptom onset [30]. The BASICS study shows that a time window of 0 to 6 h from estimated time of occlusion to IA treatment is safe in patients with a severe deficit while little can be gained in both IVT and IAT treated patients beyond the 6-h time window [2].

The main theoretical advantage of an IA approach is the variety of treatment options, which can be tailored to the individual patient. Because of the variety in approved IA treatment options and the limited number of patients, the experience with specific devices or thrombolytics varies considerably among stroke centres both within and between countries. Limiting the use of treatment options would exclude centres from participation because of lack of experience with the selected device or thrombolytic despite ample experience in the use of alternative devices or thrombolytics. New devices or thrombolytics that become available during the study may be used in the IAT arm depending on national and local approval and experience. Prior approval by the steering committee needs to be obtained.

### Trial organisation

#### Steering committee

The Steering Committee carries the ultimate responsibility for the trial. Specific tasks of the steering Committee are: (1) approval of the study protocol; (2) approval of amendments to the study protocol; (3) deciding whether or not to continue the trial based on the recommendation of the DMSB; (4) reviewing protocols for satellite studies; and (5) approval of reports and publication of the trial.

As of 18-10-2012, members of the Steering Committee are (in alphabetical order):

A. Algra*, clinical epidemiologist, University Medical Center Utrecht, Utrecht, the Netherlands;- H.J. Audebert*, neurologist, Charité Berlin, Berlin, Germany; E. Berge, neurologist, Oslo University Hospital Ullevål, Oslo, Norway; A. Ciccone, neurologist, Carlo Poma Hospital, Mantua, Italy; L.J. Kappelle*, neurologist, University Medical Center Utrecht, Utrecht, the Netherlands; M. Mazighi, interventional neurologist, Bichat University Hospital, Paris, France; P. Michel*, neurologist, University Medical Centre Vaudois, Lausanne, Switzerland; K.W. Muir, neurologist, University Medical Centre Glasgow, Glasgow, United Kingdom; V. Obach, neurologist, Clinic Hospital Barcelona, Barcelona, Spain; V. Puetz, neurologist, Dresden Stroke Centre, Dresden, Germany; W.J. Schonewille*, neurologist, St. Antonius Hospital, Nieuwegein, the Netherlands, principal investigator; J.A. Vos*, interventional radiologist, St. Antonius Hospital, Nieuwegein, the Netherlands, co-principal investigator; C.A.C. Wijman†, neurologist, Stanford University Medical Centre, Palo Alto, CA, USA; A. Zini, neurologist, St. Agostino-Estense Hospital, Modena, Italy.

* Also member of the Executive Committee

† Deceased

### Executive Committee

As of 18 October 2012, members of the Executive Committee, who are responsible for the trial on a day-to-day basis, are all members of the Steering Committee indicated with an * and E.J.R.J. van der Hoeven, radiology resident, St. Antonius Hospital, Nieuwegein, the Netherlands, coordinating investigator.

### Trial Coordination Centre

The Trial Coordination Centre is located at the Neurology department in the St. Antonius Hospital Nieuwegein in the Netherlands.

### Data Safety and Monitoring Board

An independent Data Safety and Monitoring Board, consisting of clinicians familiar with the treatment of stroke, a biostatistician and a neuro-interventionalist, has been established to monitor the progress of the trial. Details on the advice(s) of the DSMB will be notified to the Steering Committee and the METC that approved the protocol. With this notification a statement will be included indicating whether the advice will be followed.

As of 15 April 2011, members of the Data Safety and Monitoring Board, are (in alphabetical order): K.T. Hoffmann, interventional radiologist, University of Leipzig, Leipzig, Germany; P. Lyden, neurologist, Cedars-Sinai MC, Los Angeles, CA, USA (Chair); R. Raman, Biostatistician, University California, San Diego, CA, USA; D. Toni, neurologist, University ‘La Sapienza’, Rome, Italy.

### Trial status

The trial started in October 2011 in the coordinating centre, the St Antonius Hospital Nieuwegein with the inclusion of the first patient. Eleven other centres in the Netherlands, Switzerland, Czech Republic and Italy have joined since then. As of July 2013 21 patients have been randomised. In Germany, France and Spain the protocol is awaiting national ethical approval. Several other Dutch, German, French, Czech, Swiss, Italian, Spanish and Norwegian centres have indicated that they are interested in participating (http://www.basicstrial.com).

## Competing interests

The authors declare that they have no competing interests.

## Authors’ contributions

EJRJH participated in writing the protocol and is concerned with patient recruitment and data management. WJS wrote the protocol, applied for financial support and is involved in patient recruitment. AA participated in writing the protocol and is responsible for data analysis. LJK, PM and KWM participated in writing the protocol, applied for financial support and are involved in patient recruitment. HJA, EB, AC, MM, VP, MR, JAV, CACW and AZ participated in writing the protocol and are involved with patient recruitment. All authors have read and approved the manuscript.

## References

[B1] Leys D Atherothrombosis: a major health burden Cerebrovasc Dis 2001 2 1 4 11316915 10.1159/000049137

[B2] Schonewille WJ Wijman CA Michel P Rueckert CM Weimar C Mattle HP Engelter ST Tanne D Muir KW Molina CA Thijs V Audebert H Pfefferkorn T Szabo K Lindsberg PJ de Freitas G Kappelle LJ Algra A Treatment and outcomes of acute basilar artery occlusion in the Basilar Artery International Cooperation Study (BASICS): a prospective registry study Lancet Neurol 2009 8 724 730 10.1016/S1474-4422(09)70173-5 19577962

[B3] Group. TNIoNDaSr-PASS Tissue plasminogen activator for acute ischemic stroke N Engl J Med 1995 333 1581 1587 7477192 10.1056/NEJM199512143332401

[B4] Furlan A Higashida R Wechsler L Gent M Rowley H Kase C Pessin M Ahuja A Callahan F Clark WM Silver F Rivera F Intra-arterial prourokinase for acute ischemic stroke The PROACT II study: a randomized controlled trial. Prolyse in Acute Cerebral Thromboembolism. JAMA 1999 282 2003 2011 10.1001/jama.282.21.2003 10591382

[B5] Hacke W Kaste M Fieschi C von Kummer R Davalos A Meier D Larrue V Bluhmki E Davis S Donnan G Schneider D Diez-Tejedor E Trouillas P Randomised double-blind placebo-controlled trial of thrombolytic therapy with intravenous alteplase in acute ischaemic stroke (ECASS II) Second European-Australasian Acute Stroke Study Investigators. Lancet 1998 352 1245 1251 10.1016/s0140-6736(98)08020-9 9788453

[B6] Hacke W Donnan G Fieschi C Kaste M von Kummer R Broderick JP Brott T Frankel M Grotta JC Haley ECJ Kwiatkowski T Levine SR Lewandowski C Lu M Lyden P Marler JR Patel S Tilley BC Albers G Bluhmki E Wilhelm M Hamilton S Association of outcome with early stroke treatment: pooled analysis of ATLANTIS, ECASS, and NINDS rt-PA stroke trials Lancet 2004 363 768 774 15016487 10.1016/S0140-6736(04)15692-4

[B7] Hacke W Kaste M Bluhmki E Brozman M Davalos A Guidetti D Larrue V Lees KR Medeghri Z Machnig T Schneider D von Kummer R Wahlgren N Toni D Thrombolysis with alteplase 3 to 4.5 hours after acute ischemic stroke N Engl J Med 2008 359 1317 1329 10.1056/NEJMoa0804656 18815396

[B8] Ogawa A Mori E Minematsu K Taki W Takahashi A Nemoto S Miyamoto S Sasaki M Inoue T Randomized trial of intraarterial infusion of urokinase within 6 hours of middle cerebral artery stroke: the middle cerebral artery embolism local fibrinolytic intervention trial (MELT) Japan Stroke 2007 38 2633 2639 10.1161/STROKEAHA.107.488551 17702958

[B9] Lindsberg PJ Happola O Kallela M Valanne L Kuisma M Kaste M Door to thrombolysis: ER reorganization and reduced delays to acute stroke treatment Neurology 2006 67 334 336 10.1212/01.wnl.0000224759.44743.7d 16864834

[B10] Weimar C Goertler M Harms L Diener HC Distribution and outcome of symptomatic stenoses and occlusions in patients with acute cerebral ischemia Arch Neurol 2006 63 1287 1291 10.1001/archneur.63.9.1287 16966507

[B11] Macleod MR Davis SM Mitchell PJ Gerraty RP Fitt G Hankey GJ Stewart-Wynne EG Rosen D McNeil JJ Bladin CF Chambers BR Herkes GK Young D Donnan GA Results of a multicentre, randomised controlled trial of intra-arterial urokinase in the treatment of acute posterior circulation ischaemic stroke Cerebrovasc Dis 2005 20 12 17 10.1159/000086121 15925877

[B12] Arnold M Nedeltchev K Schroth G Baumgartner RW Remonda L Loher TJ Stepper F Sturzenegger M Schuknecht B Mattle HP Clinical and radiological predictors of recanalisation and outcome of 40 patients with acute basilar artery occlusion treated with intra-arterial thrombolysis J Neurol Neurosurg Psychiatry 2004 75 857 862 10.1136/jnnp.2003.020479 15146000 PMC1739049

[B13] Lindsberg PJ Mattle HP Therapy of basilar artery occlusion: a systematic analysis comparing intra-arterial and intravenous thrombolysis Stroke 2006 37 922 928 10.1161/01.STR.0000202582.29510.6b 16439705

[B14] Mattle HP Arnold M Lindsberg PJ Schonewille WJ Schroth G Basilar artery occlusion Lancet Neurol 2011 10 1002 1014 10.1016/S1474-4422(11)70229-0 22014435

[B15] Pocock SJ Clinical Trials: A Practical Approach 1983 London: Wiley

[B16] Whitehead J The Design and Analysis of Sequential Clinical Trials 1997 2 London: Wiley

[B17] del Zoppo GJ Higashida RT Furlan AJ Pessin MS Rowley HA Gent M PROACT: a phase II randomized trial of recombinant pro-urokinase by direct arterial delivery in acute middle cerebral artery stroke PROACT Investigators. Prolyse in Acute Cerebral Thromboembolism. Stroke 1998 29 4 11 10.1161/01.str.29.1.4 9445320

[B18] Khatri P Hill MD Palesch YY Spilker J Jauch EC Carrozzella JA Demchuk AM Martin R Mauldin P Dillon C Ryckborst KJ Janis S Tomsick TA Broderick JP Methodology of the Interventional Management of Stroke III Trial Int J Stroke 2008 3 130 137 10.1111/j.1747-4949.2008.00151.x 18706007 PMC3057361

[B19] Ferbert A Bruckmann H Drummen R Clinical features of proven basilar artery occlusion Stroke 1990 21 1135 1142 10.1161/01.STR.21.8.1135 2389292

[B20] Baird TA Muir KW Bone I Basilar artery occlusion Neurocrit Care 2004 1 319 329 10.1385/NCC:1:3:319 16174929

[B21] Muller R Pfefferkorn T Vatankhah B Mayer TE Schenkel J Dichgans M Sander D Audebert HJ Admission facility is associated with outcome of basilar artery occlusion Stroke 2007 38 1380 1383 10.1161/01.STR.0000260089.17105.27 17322095

[B22] Vergouwen MD Algra A Pfefferkorn T Weimar C Rueckert CM Thijs V Kappelle LJ Schonewille WJ Time is brain(stem) in basilar artery occlusion Stroke 2012 43 3003 3006 10.1161/STROKEAHA.112.666867 22989501

[B23] IMS Study Investigators Combined intravenous and intra-arterial recanalization for acute ischemic stroke: the Interventional Management of Stroke Study Stroke 2004 35 904 911 15017018 10.1161/01.STR.0000121641.77121.98

[B24] IMS II Trial Investigators The Interventional Management of Stroke (IMS) II Study Stroke 2007 38 2127 2135 17525387 10.1161/STROKEAHA.107.483131

[B25] Lewandowski CA Frankel M Tomsick TA Broderick J Frey J Clark W Starkman S Grotta J Spilker J Khoury J Brott T Combined intravenous and intra-arterial r-TPA versus intra-arterial therapy of acute ischemic stroke: Emergency Management of Stroke (EMS) Bridging Trial Stroke 1999 30 2598 2605 10.1161/01.STR.30.12.2598 10582984

[B26] Broderick JP Palesch YY Demchuk AM Yeatts SD Khatri P Hill MD Jauch EC Jovin TG Yan B Silver FL von Kummer R Molina CA Demaerschalk BM Budzik R Clark WM Zaidat OO Malisch TW Goyal M Schonewille WJ Mazighi M Engelter ST Anderson C Spilker J Carrozzella J Ryckborst KJ Janis LS Martin RH Foster LD Tomsick TA Interventional Management of Stroke (IMS) III Investigators: Endovascular therapy after intravenous t-PA versus t-PA alone for stroke N Engl J Med 2013 368 893 903 10.1056/NEJMoa1214300 23390923 PMC3651875

[B27] Nogueira RG Lutsep HL Gupta R Jovin TG Albers GW Walker GA Liebeskind DS Smith WS Trevo versus Merci retrievers for thrombectomy revascularisation of large vessel occlusions in acute ischaemic stroke (TREVO 2): a randomised trial Lancet 2012 380 1231 1240 10.1016/S0140-6736(12)61299-9 22932714 PMC4176618

[B28] Saver JL Jahan R Levy EI Jovin TG Baxter B Nogueira RG Clark W Budzik R Zaidat OO Solitaire flow restoration device versus the Merci Retriever in patients with acute ischaemic stroke (SWIFT): a randomised, parallel-group, non-inferiority trial Lancet 2012 380 1241 1249 10.1016/S0140-6736(12)61384-1 22932715

[B29] Shaltoni HM Albright KC Gonzales NR Weir RU Khaja AM Sugg RM Campbell MS Cacayorin ED Grotta JC Noser EA Is intra-arterial thrombolysis safe after full-dose intravenous recombinant tissue plasminogen activator for acute ischemic stroke? Stroke 2007 38 80 84 10.1161/STROKEAHA.107.486126 17122433

[B30] Nogueira RG Liebeskind DS Sung G Duckwiler G Smith WS Predictors of good clinical outcomes, mortality, and successful revascularization in patients with acute ischemic stroke undergoing thrombectomy: pooled analysis of the Mechanical Embolus Removal in Cerebral Ischemia (MERCI) and Multi MERCI Trials Stroke 2009 40 3777 3783 10.1161/STROKEAHA.109.561431 19875740

